# Budesonide/formoterol for pediatric asthma: bridging the gap between evidence and practice in Maintenance and Reliever Therapy

**DOI:** 10.3389/fmed.2025.1712238

**Published:** 2025-10-30

**Authors:** Fu-xiang Tian, Xiu-ping Wang, Dong-mei Wang

**Affiliations:** Department of Pediatrics, Yan’an People’s Hospital, Yan’an, China

**Keywords:** pediatric asthma, budesonide, formoterol, asthma management, treatment adherence, personalized medicine

## Abstract

Budesonide/formoterol is established as a foundational therapy for moderate-to-severe pediatric asthma, with extensive evidence supporting its efficacy in both maintenance and Maintenance and Reliever Therapy regimens. Its dual pharmacologic profile provides distinct advantages in exacerbation reduction and symptom control. However, clinical implementation faces persistent challenges, including diagnostic uncertainty in young children, technical difficulties in device use, variable adherence, and perceptual barriers among clinicians and caregivers. Future advancement requires targeted research into phenotype-specific treatment responses, integration of digital health technologies for personalized management, and systematic reform in education and policy. A multidisciplinary effort is essential to achieve more precise, effective, and sustainable asthma control in the pediatric population.

## 1 Introduction

### 1.1 Epidemiology and disease burden of pediatric asthma

Asthma is a predominant chronic respiratory condition among children globally, with increasing prevalence noted in numerous regions (1, 2). This disease imposes a considerable burden on healthcare systems, contributes to missed school days, and diminishes the quality of life of affected children (2, 3). Recurrent symptoms and exacerbations also place significant emotional and financial strain on families, underscoring the critical need for effective and sustainable management strategies (3, 4).

### 1.2 Stepwise management strategy in pediatric asthma

International guidelines, including the Global Initiative for Asthma (GINA), recommend a stepwise approach to asthma treatment (5, 6). This strategy involves adjusting therapeutic intensity according to the level of symptom control and exacerbation risk (5, 6). For children with moderate-to-severe asthma who experience persistent symptoms despite low- to medium-dose inhaled corticosteroids (ICS), the introduction of a long-acting beta2-agonist (LABA) in combination with ICS is advised (7). This approach underscores the importance of ICS + LABA therapy in achieving improved asthma control.

### 1.3 Pharmacological characteristics and theoretical advantages of budesonide/formoterol

Budesonide/formoterol combines budesonide, an inhaled corticosteroid with a favorable pharmacokinetic profile, and formoterol, a long-acting beta2-agonist characterized by its rapid onset of action (8–10). This combination offers complementary mechanisms: budesonide targets airway inflammation, while formoterol provides prolonged bronchodilation (10). Their concurrent rapid onset enables not only maintenance treatment but also effective relief of acute symptoms, forming the basis for the Maintenance and Reliever Therapy (MART) regimen (11, 12). This dual functionality supports flexible dosing and has the potential to enhance treatment adherence.

It is important to note that the budesonide/formoterol combination is available in several inhaler devices, such as the Turbuhaler^®^, Spiromax^®^, and Forspiro^®^ (13, 14). These delivery systems possess distinct technical characteristics, but they share the core pharmacological combination of budesonide and formoterol (15, 16). This article focuses on the clinical evidence and therapeutic principles of the budesonide/formoterol combination as a pharmacological class, while specific device-related considerations are addressed in the context of practical implementation.

### 1.4 Objective and structure of this perspective

This perspective aims to critically evaluate the current evidence regarding the use of budesonide/formoterol in pediatric asthma management. Rather than providing a systematic review, it seeks to identify key challenges—such as safety concerns in long-term use, appropriate patient selection, and practical barriers to implementation—and to propose informed directions for future clinical research and guideline development. The structure reflects a progression from established concepts to emerging issues and forward-looking recommendations.

## 2 Current therapeutic status: an evidence-based perspective

### 2.1 Efficacy evidence

#### 2.1.1 Evidence for maintenance therapy

Multiple randomized controlled trials (RCTs) and meta-analyses have established the efficacy of budesonide/formoterol as maintenance therapy in pediatric asthma. These studies consistently demonstrate its superiority to ICS monotherapy in improving lung function, reflected by significant increases in forced expiratory volume in 1 s (17, 18). Additionally, budesonide/formoterol has shown enhanced control of both daytime and nocturnal symptoms and a reduced reliance on short-acting beta2-agonists (SABA) for symptom relief, indicating effective overall disease management (18, 19).

#### 2.1.2 Evidence for MART

A substantial body of evidence supports the use of budesonide/formoterol within the MART regimen in pediatric patients. Clinical trials specifically highlight its capacity to significantly reduce the frequency of asthma exacerbations compared to fixed-dose ICS-LABA combinations with separate SABA relievers (10, 20, 21). This effect is largely attributed to the complementary pharmacology of budesonide and formoterol, which together provide both sustained anti-inflammatory support and rapid bronchodilation, enabling early intervention during symptom worsening and preventing progression to full exacerbations (20, 22).

The MART regimen employs a flexible dosing strategy, combining a fixed maintenance dose (e.g., 1-2 inhalations of budesonide/formoterol twice daily) with as-needed reliever use (1 additional inhalation for acute symptom relief) (23). The total daily dose must be tailored to the patient’s asthma control and exacerbation frequency, while strictly adhering to the maximum licensed daily limit (e.g., 8 inhalations per day for the 160/4.5 μg formulation in pediatric patients) (9, 24). Successful implementation requires comprehensive education to ensure that patients and caregivers can distinguish symptoms requiring reliever doses and understand the core principle of flexible dosing within a predefined safety framework (9, 24–26).

### 2.2 Safety profile

Long-term studies support the acceptable safety profile of budesonide/formoterol in children. Assessments of growth parameters, including annual height velocity and adult height, indicate minimal and generally non-significant effects from budesonide at conventional therapeutic doses (27, 28). Similarly, evaluations of hypothalamic- pituitary-adrenal axis function have not revealed clinically relevant suppression (10). Among available ICS/LABA combinations, budesonide/formoterol is one of the most extensively studied in pediatric populations, with a well-documented and favorable long-term safety record (10, 27, 28).

### 2.3 Position in clinical guidelines

Based on this evidence, budesonide/formoterol is endorsed in several major international and national guidelines for pediatric asthma management. The GINA recommends budesonide/formoterol MART as a preferred treatment option for children aged 6 and above at treatment Step 3 or higher, particularly those whose symptoms remain uncontrolled despite medium-dose ICS (5). This recommendation emphasizes the regimen’s value in reducing exacerbation risk. Parallel guidance is provided by the Chinese Guidelines for Childhood Asthma, which recognize ICS-LABA combination therapy as a standard approach for school-aged children and adolescents with moderate-to-severe asthma that is inadequately controlled on medium-dose ICS, explicitly supporting MART strategy in eligible patients (29, 30). It is important to note that successful implementation requires not only meeting the clinical criteria but also ensuring the child’s ability to use the inhaler correctly and the family’s understanding of the MART regimen.

## 3 Clinical challenges and practical implementation barriers

### 3.1 Diagnostic accuracy and patient stratification

The initial challenge in utilizing budesonide/formoterol effectively lies in the accurate diagnosis of asthma and subsequent patient stratification. Particularly in preschool-aged children, distinguishing true asthma from transient viral-induced wheezing remains diagnostically challenging (31–33). This differentiation is crucial, as it determines the appropriateness of long-term controller therapy (31, 32). Furthermore, imprecise assessment of disease severity—a prerequisite for stepwise treatment allocation—may result in either inappropriate withholding of ICS/LABA therapy in eligible patients or its unnecessary use in mild cases, potentially altering risk-benefit considerations (32, 33).

### 3.2 Technical challenges in drug delivery

Optimal therapeutic outcomes with budesonide/formoterol Turbuhaler^®^ are contingent upon achieving adequate inhaler technique (34). The device requires a rapid and deep inspiratory effort to disaggregate and deliver the powdered medication (34). Pediatric patients, especially those under age 8, often lack the coordination and inspiratory force to use dry powder inhalers correctly (35). Suboptimal inhalation technique significantly diminishes lung deposition and clinical efficacy (36). This necessitates repeated training sessions and frequent monitoring, imposing substantial demands on healthcare providers’ time and clinic resources.

### 3.3 Adherence and long-term management barriers

Sustaining treatment adherence represents a fundamental challenge in chronic asthma management (37). Discontinuation of therapy following symptom improvement is frequently observed, leading to uncontrolled inflammation and increased exacerbation risk (38). Additional systemic barriers include fragmented care between clinical and community settings, logistical challenges in maintaining regular follow-up, and socioeconomic factors such as treatment costs (37, 39). These elements collectively compromise the long-term effectiveness of budesonide/formoterol, despite its proven efficacy in controlled trial settings.

### 3.4 Knowledge gaps and perceptual barriers

The implementation of evidence-based strategies, particularly MART, is often hindered by perceptual and educational barriers. Some practitioners remain cautious due to unfamiliarity with MART principles or concerns regarding off-label dosing in younger children (40, 41). Concurrently, parental apprehensions regarding inhaled corticosteroid safety—often termed “corticophobia”—may lead to deliberate dose reduction or therapy avoidance (42, 43). Bridging these gaps requires targeted educational initiatives for both healthcare providers and families to align perceptions with current evidence and guidelines (40, 44).

## 4 Future directions and strategic development

### 4.1 Evidence generation and research priorities

Future research must address critical evidence gaps to optimize budesonide/formoterol’s application in pediatric asthma. A primary focus should be placed on conducting large-scale, methodologically robust RCTs specifically targeting preschool populations, where diagnostic ambiguity often complicates treatment decisions (45, 46). Research should also investigate differential treatment responses across various asthma endotypes—particularly T2-high versus T2-low phenotypes—to establish predictive biomarkers for therapy selection (33, 45). Additionally, comprehensive longitudinal studies are essential to further elucidate the long-term safety profile, with specific attention to potential effects on neurodevelopmental trajectories and final adult height (45, 46).

### 4.2 Advancing personalized treatment approaches

In an era of expanding biological therapies, defining budesonide/formoterol’s role requires a more nuanced understanding of patient-specific factors. Future efforts should focus on identifying clinical and biomarker profiles that predict optimal response to budesonide/formoterol therapy (47, 48). Systematic investigation of biomarkers such as fractional exhaled nitric oxide and peripheral blood eosinophil counts could facilitate more precise patient selection and dosing strategies (47, 49). This approach would enable stratification of patients who benefit most from budesonide/formoterol versus those who might require alternative therapies, thereby advancing personalized treatment protocols (49, 50).

### 4.3 Technological innovations in disease management

Digital health technologies offer transformative potential for addressing persistent management challenges. Integration of smart inhaler devices with embedded sensors can provide objective data on medication adherence, inhalation technique, and environmental exposures (51). These data systems should be coupled with interoperable digital platforms that enable remote monitoring, automated medication reminders, and telehealth consultations (52). Such technological integration can bridge the gap between clinical encounters and daily disease management, facilitating more responsive and data-driven asthma care (53).

### 4.4 Health system and educational initiatives

Strategic health system reforms are necessary to maximize budesonide/formoterol’s therapeutic potential. Policy review should focus on expanding insurance coverage for MART regimens to improve treatment accessibility and reduce financial barriers (54, 55). Concurrently, enhanced professional education programs must be developed for frontline healthcare providers, emphasizing practical skills in device training, patient selection for MART, and addressing corticosteroid-related concerns (55, 56). Complementary public health initiatives should develop validated educational resources to improve health literacy and self-management skills among patients and caregivers, ultimately supporting more effective implementation of evidence-based asthma management strategies (56, 57).

## 5 Summary

Budesonide/formoterol represents a well-established therapeutic option for moderate-to-severe pediatric asthma, with a substantial evidence base demonstrating its efficacy in both maintenance and MART regimens. Its dual pharmacological profile offers unique benefits for exacerbation reduction and symptom control. Nevertheless, several implementation barriers persist, including diagnostic uncertainties across developmental stages, technical challenges associated with inhaler use, variable treatment adherence, and knowledge gaps among healthcare providers and families regarding its optimal application.

Addressing these challenges requires a coordinated multidimensional approach. Future progress depends on generating more precise evidence through phenotype-stratified clinical trials, particularly in preschool populations. Furthermore, integrating digital health technologies for monitoring and adherence support, coupled with enhanced educational initiatives for both medical professionals and caregivers, will be crucial. Through these concerted efforts, the pediatric asthma community can advance toward more personalized, effective, and safer asthma management strategies that maximize therapeutic outcomes while minimizing risks.

## Data Availability

The original contributions presented in this study are included in this article/supplementary material, further inquiries can be directed to the corresponding author.
